# Nasal filling guided by high frequency ultrasound: Reducing risks

**DOI:** 10.1111/jocd.16430

**Published:** 2024-07-03

**Authors:** Bruna Souza Felix Bravo, Raquel De Melo Carvalho, Mariana Calomeni, Camila Silveira Nobre, Gabriel Leal Telino Alves, Leonardo Gonçalves Bravo, Thamires Silva Cavalcante de Almeida

**Affiliations:** ^1^ Dermatology Department Bravo Private Clinic Rio de Janeiro Brazil

**Keywords:** high frequency ultrasound, hyaluronic acid, nasal filling, rhinoplasty

## Abstract

**Background:**

Given the significant increase in the quantity of cosmetic procedures utilizing hyaluronic acid fillers, including in the nasal region, the initial evaluation of patients using high frequency ultrasound becomes a crucial instrument in evaluating and handling nonsurgical rhinoplasty.

**Aims:**

The aim of this article is to introduce an assessment methodology for nasal filling guided by high frequency ultrasound.

**Patients/Methods:**

A prospective and single‐center study was conducted with 12 Latin American patients. The patients underwent nasal filling with hyaluronic acid following high power ultrasound mapping.

**Results and Conclusions:**

In the evaluation of the GAIS scale, all patients reported improvement with the treatment. No infections, nodules, ischemia, or other relevant adverse effects were noted. Real‐time ultrasound‐guided filler techniques have been developed to reduce the risk of vascular compromise, confirming the distribution pattern of blood vessels. It's also crucial to visualize the cannula at the same moment as the vessels, even if the previous vascular mapping was performed. Therefore, the utilization of high frequency ultrasound can act as a pivotal tool in augmenting procedure safety.

## 1 INTRODUCTION

Nasal morphology plays a crucial role in facial proportions. A well‐structured nose elevates the beauty of the whole face. At present, rhinoplasty stands as the gold standard for rectifying nasal aesthetic imperfections, offering enhancements with a remarkable level of precision.^1^ Therefore, rhinoplasty continues to rank among the top five most sought‐after cosmetic surgeries.^2^

However, surgery may not be the preferred approach in some contexts, such as treating discreet imperfections or cases of dissatisfaction with a previous surgical procedure. In these cases, nasal filling with hyaluronic acid has proven to be a safe, less invasive alternative with satisfactory results and a shorter recovery time.^2^, ^3^

Accurate assessment of facial and nasal parameters, along with the suitable selection of the product utilized for nasal filling, is essential to achieve satisfactory and safe results with the procedure. Data from plastic surgery societies in the United States show a clear trend of decrease in the popularity of surgical rhinoplasty coincides with a simultaneous rise in the utilization of fillers for nasal corrections.^4^

Given the substantial increase in the number of aesthetic procedures with non‐permanent fillers, including the nasal region, the initial evaluation of patients using high frequency ultrasound becomes fundamental to provide greater safety for both patients and professionals.

When analyzing the anatomical locations on the face where injections most frequently resulted in visual impairment related to injection, in various independent studies, the nose was consistently identified as the most prevalent area of concern, followed by the glabella and forehead.^5^ The heightened risk of visual impairment associated with injection is logical due to these regions having their arterial blood supply as direct extensions of the ophthalmic artery, the vessel responsible for supplying arterial blood to the retina.^6^

Therefore, a precise clinical and ultrasound evaluation is of great importance for determining.

The ideal amount of filling, the appropriate injection site, and the presence of anatomical variations in order to avoid adverse events that may negatively affect patients.

The objective of this article was to illustrate a method for assessing nasal filling to reduce vascular damage during the injection of soft tissue fillers, validating the pattern of blood vessel distribution through ultrasound in Latin American patients.

## 2 METHODS

### 2.1 Study design

A prospective and single‐center study was conducted with 12 Latin American patients—10 women and 2 men—in June 2023 at a private clinic in the City of Rio de Janeiro. Their ages ranged from 27 to 59 years, and they had Fitzpatrick skin phototypes II–IV. The patients underwent nasal filling with hyaluronic acid following high‐power ultrasound mapping. Data were meticulously recorded on dedicated forms designed for data collection.

The study adhered to ethical parameters outlined in the Helsinki Declaration, and informed consent was obtained from all participating patients. Exclusions comprised individuals with a history of prior nasal filling or rhinoplasty involving implants and autologous grafts, as obtaining precise measurements in such cases is challenging. Additional exclusion criteria encompassed skin infections, inflammation, scars, pigmentary disorders or implants in the treatment areas, autoimmune diseases, the use of immunosuppressive medications, as well as pregnancy or breastfeeding.

### 2.2 Data analysis

Standardized images were obtained using the Vectra®‐H2 3D Imaging System software (Canfield Scientific, Parsippany, NJ, USA) as static photos for clinical and volumetric assessment before and after the procedure. Adverse effects were evaluated during treatment and follow‐up. Patient age, gender, and nasal changes were recorded for all individuals enrolled at the beginning of the study.

Participants were questioned about possible adverse events through a standardized questionnaire, and a dermatologist visually examined the treatment area during the follow‐up consultation.

The clinical analysis involved photographic assessment by two blinded investigators, and a consensus subjective evaluation was conducted by physicians (GAIS) using the 5‐point Global Aesthetic Improvement Scale.

### 2.3 Ultrasonographic analysis

All ultrasonographic measurements were performed by the same investigator (M.C.), a radiologist and dermatologist with extensive experience in dermatological ultrasonography, ensuring consistency throughout the study.

Patients selected to undergo the procedure underwent high frequency ultrasound using the Logiq (GE Healthcare, Milwaukee, WI), with predefined settings and a compact linear transducer, “hockey stick” model, with a variable frequency of L8‐18 MHz, always applying its maximum frequency of 18 MHz. Ultrasonographic images were recorded in grayscale, in addition to Color Doppler and Power Doppler modes.

Study participants were seated in a tilted position (45°C) for all measurements taken. The transducer was placed at a thick layer of ultrasound contact gel. (Aquasonic Clear Ultrasound Gel, Parker Laboratories Inc., Fairfield, NJ, USA), without direct contact with the skin, allowing visualization and measurement of the tissue layer without skin pression and compression variation (Figures 1, 2, 3).

**FIGURE 1:**
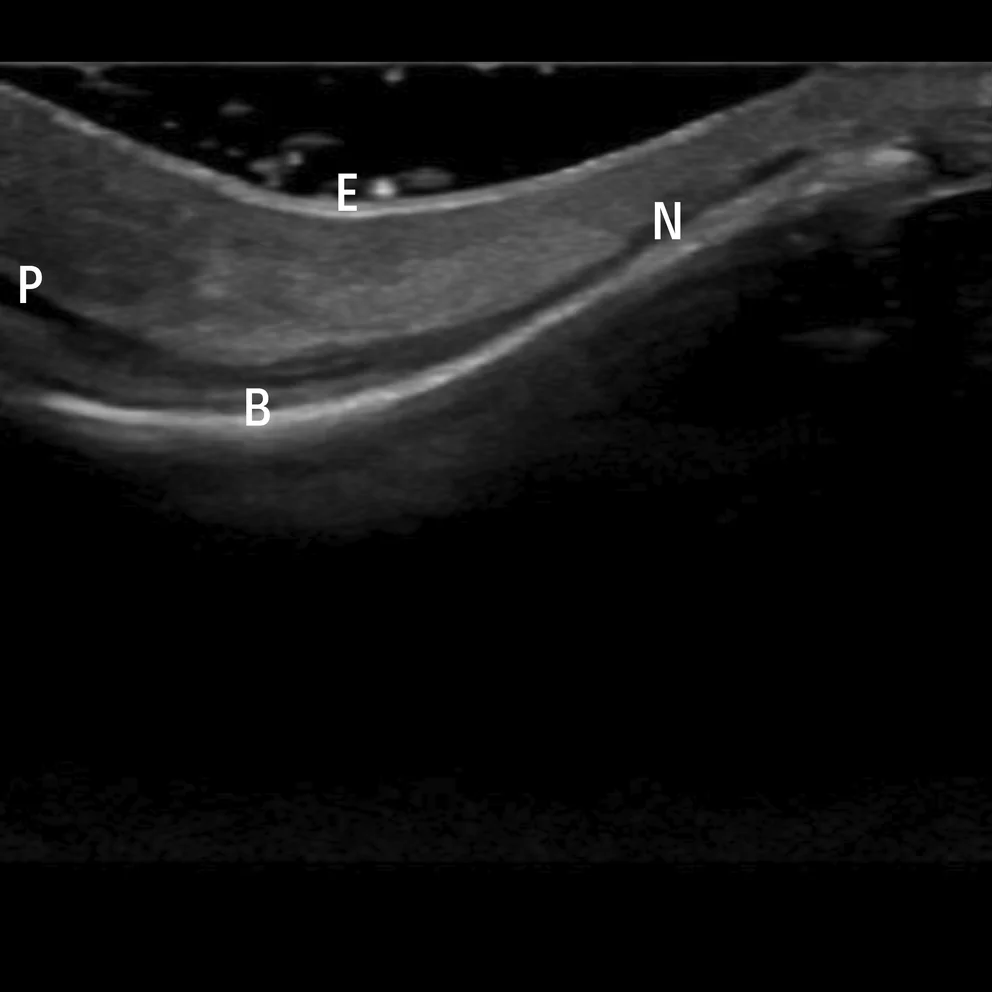
Ultrasound scan: anatomy of the radix. B, bone – nasal bone; E, epidermis; N, nasalis muscle; P, procerus muscle.

**FIGURE 2:**
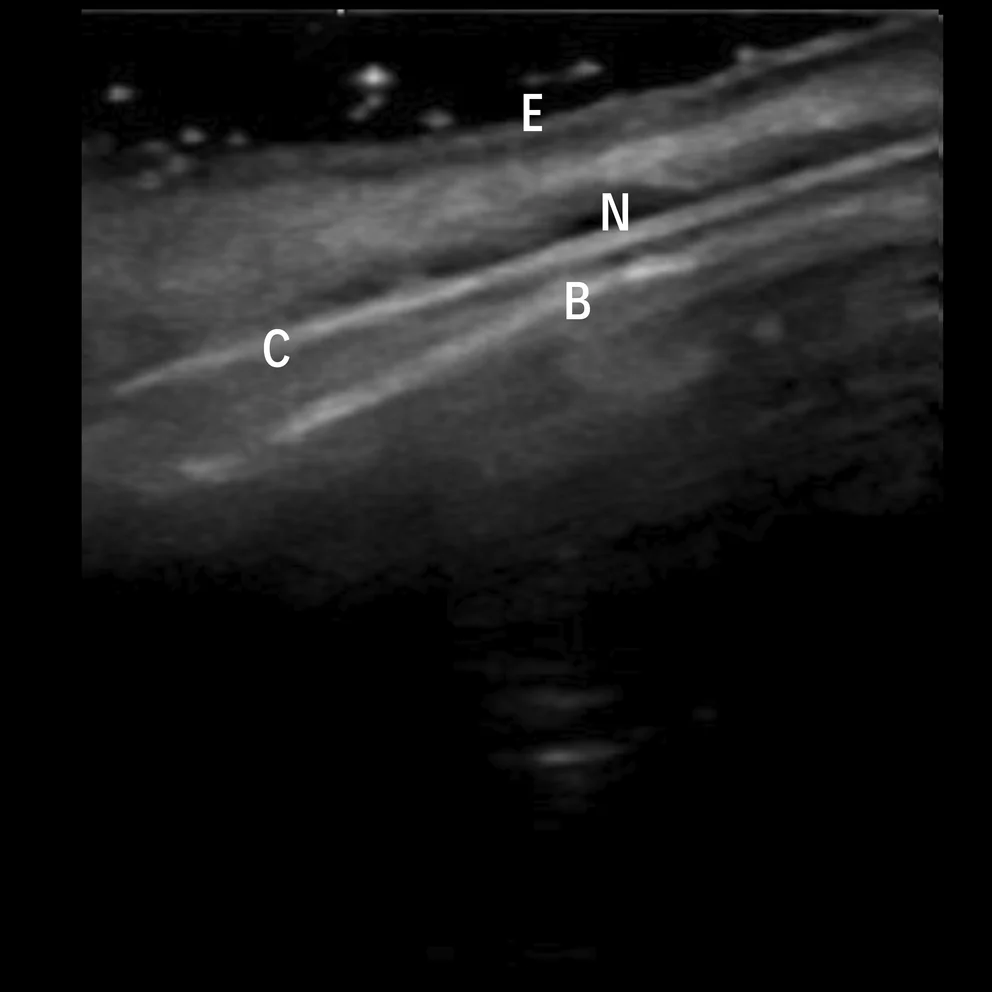
Ultrasound scan: anatomy of the radix. B, bone – nasal bone; C, cannula; E, epidermis; N, nasalis muscle.

**FIGURE 3:**
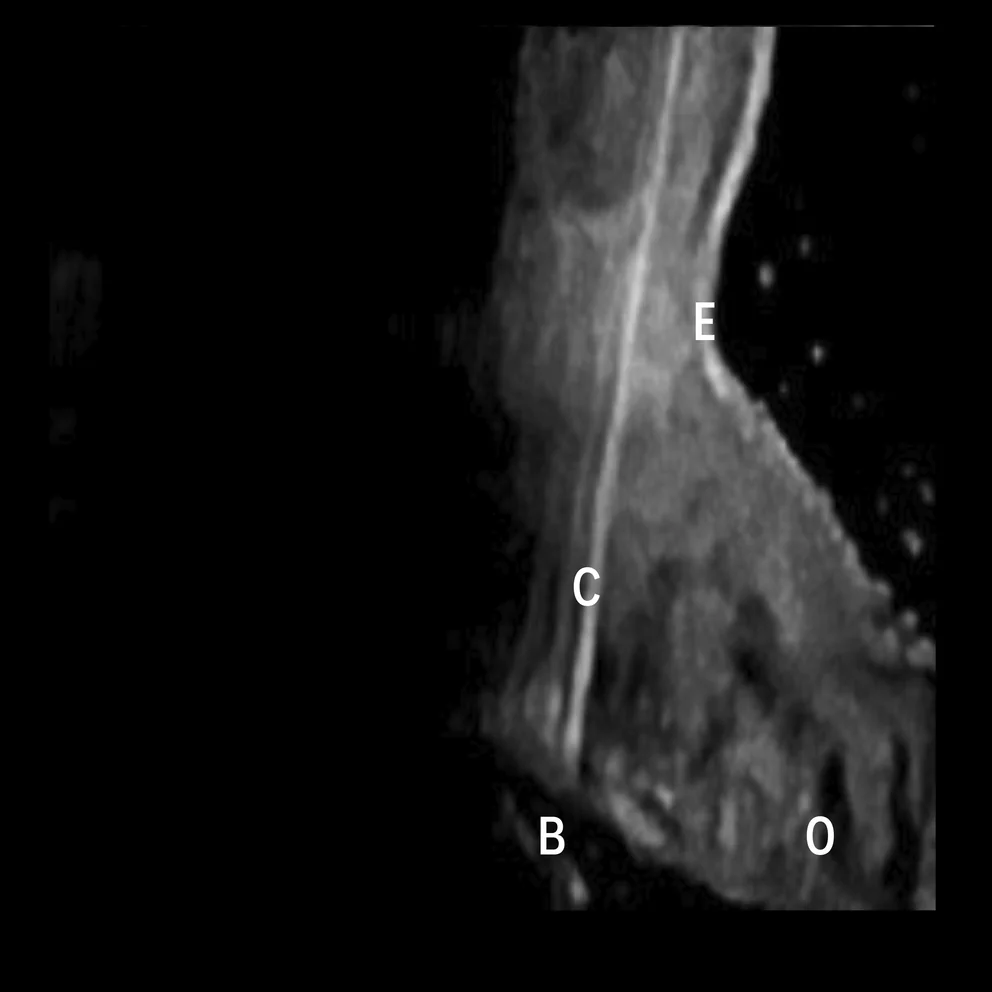
Ultrasound scan: anatomy of the columela. B, bone – nasal spine; C, cannula; E, epidermis; O, orbicular oris muscle.

The ultrasonographic examination was performed at four anatomical points: radix, rhinion, tip, and columella. The radix is a concavity that corresponds to the junction of the nasal and frontal bones, inferior to the glabella, at the level of the upper eyelid creases. The rhinion refers to the transition between the nasal bone and the upper nasal cartilages. The tip is the most projected portion of the nasal tip, anteriorly. The columella corresponds to the cartilaginous nasal portion located between the nostrils.

### 2.4 Injection technique

All cases were under the care of an experienced dermatologist (BB) with 18 years of expertise in nonsurgical rhinoplasty using hyaluronic acid fillers. The procedural protocol included cleansing the skin with a 2% alcoholic chlorhexidine solution, followed by local anesthesia using lidocaine without vasoconstrictor in the infratip region beneath the nasal tip. The entry point was skillfully made using a 21G with a length of 25 mm needle for the subsequent insertion of a 22G cannula with a length of 50 mm.

### 2.5 Description of the injection

The chosen product for filling was Rennova® Lift Plus Lido, a cross‐linked hyaluronic acid filler produced using the XPMTechTM technology, featuring a concentration of 25 mg/mL of hyaluronic acid and high viscoelasticity. Therefore, it can be particularly suitable for use in the nose.

The quantity of hyaluronic acid administered in each specific area varied based on the individual case and the corresponding application plan, with a maximum limit of 1 mL per patient. Subsequent to the injection process, the product was meticulously adjusted manually, and a small sterile dressing was exclusively applied to the puncture sites, allowing the identification of potential early complications.

## 3 RESULTS

Standardized images were obtained immediately after the procedure using Vectra 3D Imaging System (Canfield). All patients (12) underwent nasal hyaluronic acid filling guided by high frequency ultrasound, ensuring safety in applications with no perceived adverse effects. (Figures 4, 5, 6).

**FIGURE 4:**
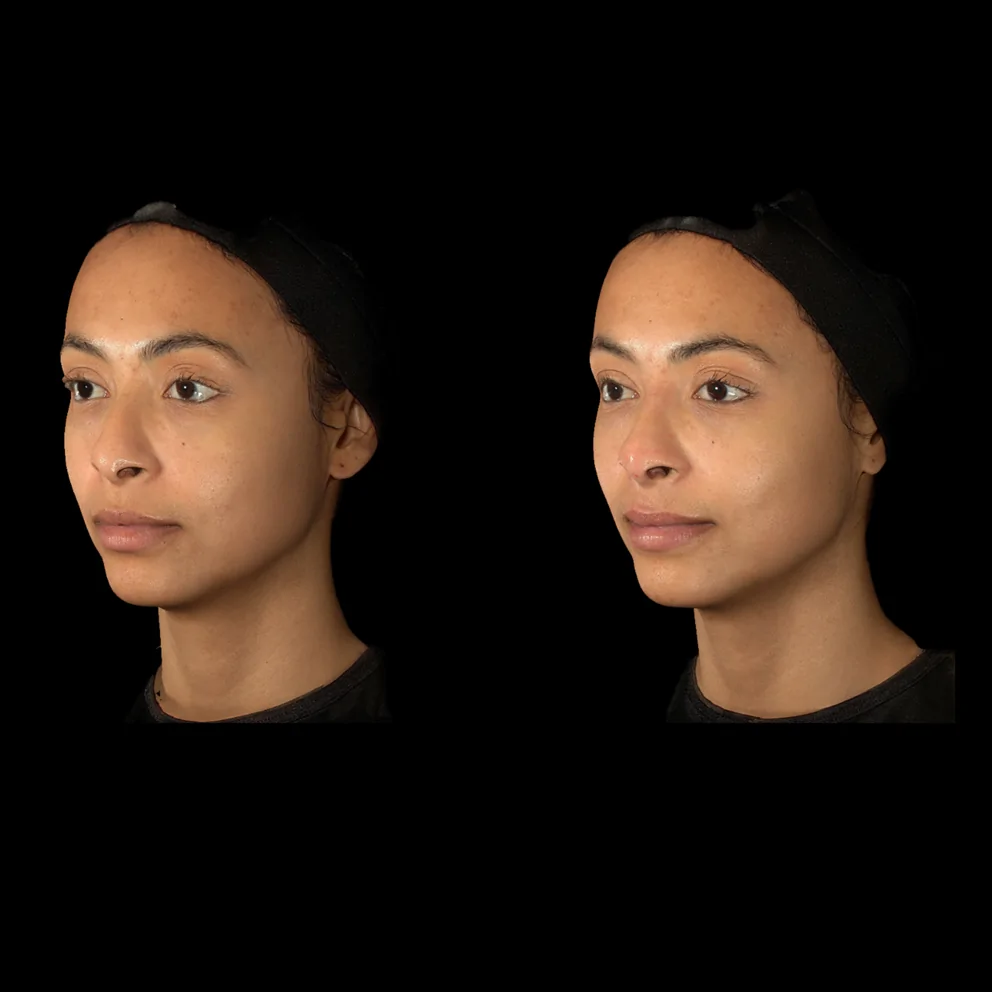
Female, 27 years. The 45 degree angle before and after nasal filler injections.

**FIGURE 5:**
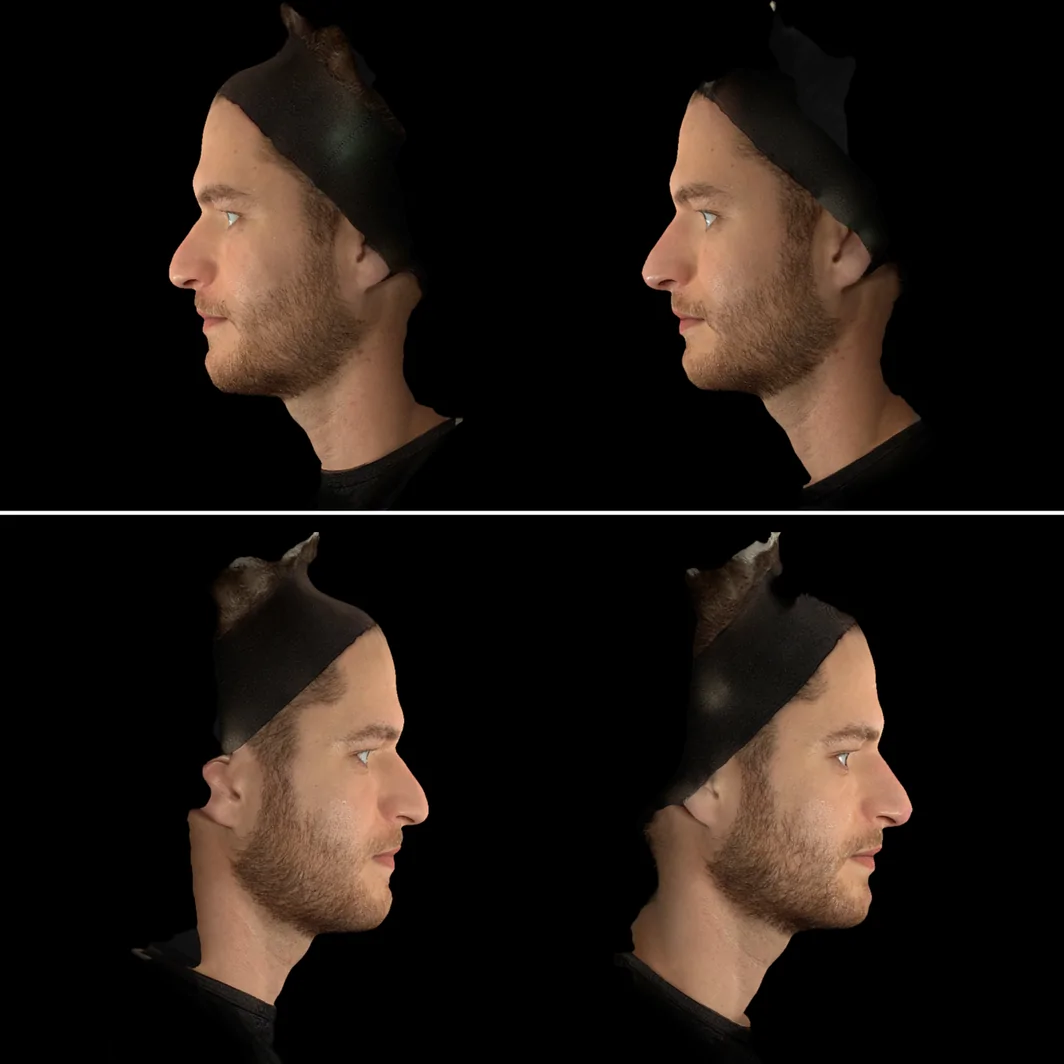
Male, 29 years. The profile view before and after nasal filler injections.

**FIGURE 6:**
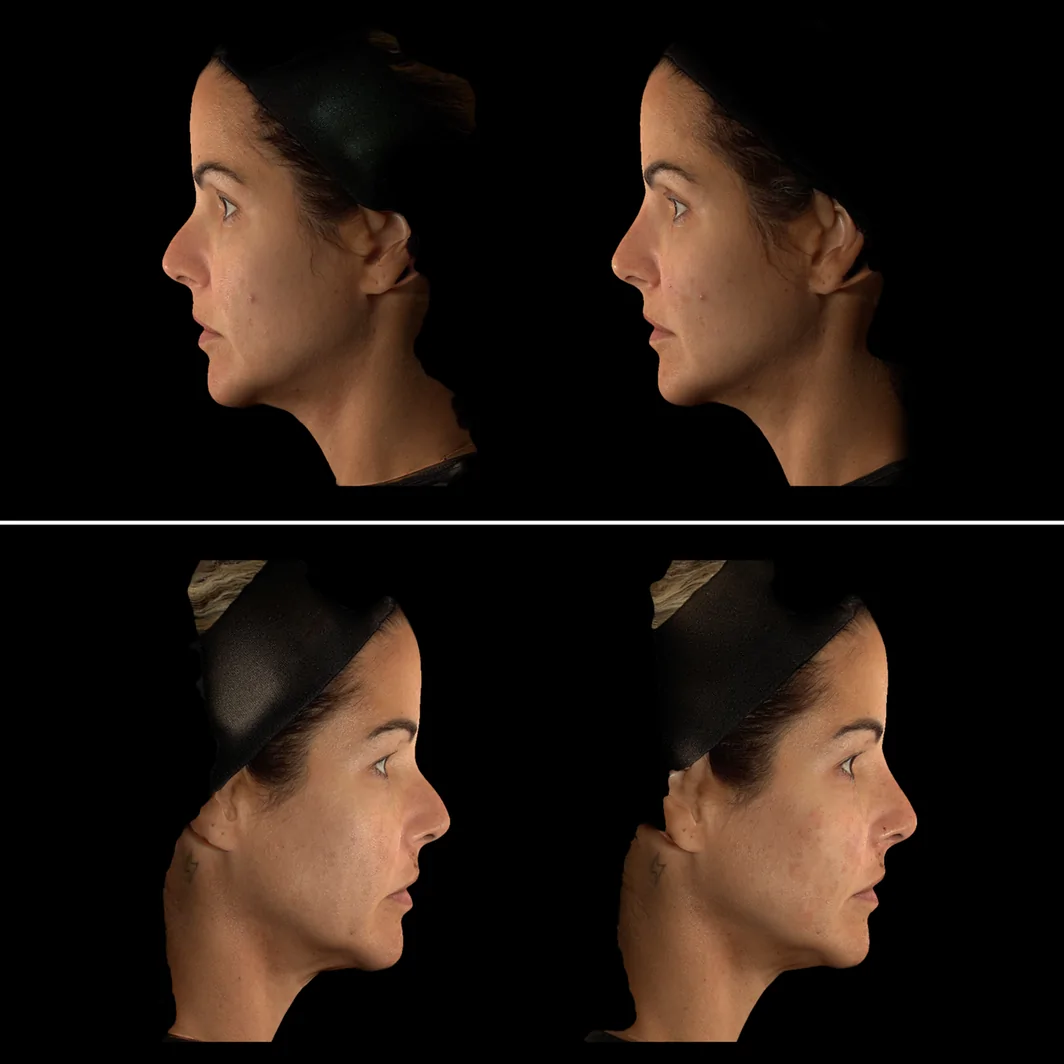
Female, 44 years. The profile view before and after nasal filler injections.

Treatment response rates remained high until the completion of the study, with subjective patient assessment conducted through the Global Aesthetic Improvement Scale (GAIS) at the end of 6 months, receiving a score of 1 (very much improved) in 8 patients and a score of 2 (improved) in 4 patients. In this study, the treatment effects persisted until the conclusion of the study, yet long‐term follow‐up will be essential to offer a comprehensive evaluation of durability. No additional applications or protocol deviances regarding volume or dilution were required. No infections, nodules, ischemia, or other relevant adverse effects were noted.

## 4 DISCUSSION

The nose is a three‐dimensional structure situated in the middle third of the face. Anatomically, the nose extends inferiorly from the forehead and can be subdivided into three portions. The upper portion includes the nasal bones and the frontal process of the maxilla; the middle portion is composed of the lateral nasal cartilages; and the lower portion encompasses the inferior nasal spine and the larger alar cartilages. Additionally, the anatomical connection between the cartilages is primarily maintained by the piriform and interalar ligaments.^7^

Various elements and external reference points must be considered when analyzing the characteristics and potential changes in the nose. The nose can be subdivided into the radix, nasal base (deepest part), rhinion (bony and cartilaginous junction), supra tip (point immediately above the nasal tip), and nasal tip (image 2). Based on these anatomical references, the nasal tip emerges as the most prominent section of the nose, while the nasal pyramid encompasses the nostrils, columella, and nasal wings. The lower segment of the columella is identified as the subnasal region. Furthermore, it's crucial to recognize that the nasal wings form the sides of the nasal tip and join the tip lobule with the facial skin.^8^

The nose layer arrangement, from the outer to inner, consist in skin, subcutaneous tissue, superficial fatty, deep areolar tissue, periosteum or perichondrium, besides, bone or cartilage, which could range depends on the anatomical portion.

The nasal region's vascularization involves a sophisticated network of blood vessels derived from both the internal and external carotid arteries, creating a huge amount of anastomosis. This intricate system ensures adequate blood supply to various nasal structures, supporting their function and viability. One crucial anastomosis exists between the internal and external carotid systems through the ophthalmic artery, a branch of the internal carotid artery. The ophthalmic artery gives off an important branch, called dorsal nasal artery, which communicates with branches of the facial artery, ensuring continuous blood flow to the nasal dorsum.

Anastomoses between the superior labial artery, a branch of the facial artery, and the greater palatine artery, a branch the internal maxillary artery, provide collateral circulation to the anterior nasal septum and nasal ala.

When fillers are inadvertently intra‐arterial injected, particularly around the nose, they might reach branches of the ophthalmic artery, causing retrograde embolization. This embolization can lead to central retinal artery occlusion, resulting in sudden vision loss due to retinal ischemia.

At the nasal radix and at the mid‐nasal dorsum, the arterial vasculature was located subdermally within the superficial fatty layer.

The increasing preference for nonsurgical rhinoplasty using hyaluronic acid among both patients and physicians can be ascribed to several factors. These include reduced costs, a brief recovery period, reversibility (when performed with HA fillers), immediate and visible results, and the perceived safety associated with a semi‐invasive procedure as opposed to surgery.^7^, ^9^, ^10^, ^11^, ^12^, ^13^

Complications in nonsurgical rhinoplasty can be broadly classified into factors associated with the patient, the physician, or the product. Prudent patient selection, grounded on realistic expectations, along with the ability to distinguish cases suitable for nonsurgical intervention from those more apt for conservative or surgical approaches, clinical contraindications, and a thorough psychological assessment, collectively contribute to minimizing risks stemming from patient‐related factors.

In a retrospective study involving 500 patients undergoing nonsurgical rhinoplasty, Harb et al. (2010) observed immediate arterial occlusion in 24 patients, manifested by painless hypochromia of the skin at the time of treatment, typically in an irregular distribution either at the injection site or distant from it. In these instances, the treatment was discontinued, and prompt management of impending ischemia was initiated. Out of the 24 cases of vascular compromise, the nasal tip injection site (16 patients) was the area most frequently affected. Fourteen patients experienced a spontaneous return to adequate perfusion following vigorous skin massage and the application of warm compresses. Ten patients received immediate hyaluronidase infiltration treatment and subsequently achieved complete recovery without necrotic complications.^9^

In another case series involving 295 patients, Rivkin et al. 2010 reported two cases of skin necrosis and two cases of visual irregularities following nasal filling.^14^

Clinical factors, including injection technique, a thorough comprehension of local anatomy, proficiency in recognizing potential complications, and skill in effectively managing these complications, are the principal determinants of both success and complication rates. Additionally, factors related to the product, including viscosity, the extent of cross‐linking, comprehensive anesthesia, and reversibility, also wield significant influence over the ultimate outcome.^9^ Injecting the product too deeply can alter normal nasal anatomy and, in turn, complicate additional procedures, particularly surgical rhinoplasty. A very superficial injection (e.g., intradermal or subdermal) primarily poses the risk of vascular occlusion or skin necrosis.

Moreover, in patients with a history of surgical rhinoplasty, there is frequently a disturbance in the blood supply to the columella, potentially heightening the risk of vascular insufficiency and skin necrosis. These risks underscore the necessity for professionals conducting nonsurgical rhinoplasty to possess a thorough understanding of nasal anatomy and aesthetics.^9^

In a clinical trial conducted with high frequency ultrasound in 60 patients, Alfertshofer et al. (2022)^6^ observed a five‐layer arrangement in the middle nasal dorsum in 100% of the investigated cases, while it was absent at the nasal root and tip. The five‐layer configuration demonstrated an average extent ranging from 26.7% to 67.5% in relation to nasal length. Nasal arteries were found to run superficially in 91.7% of all cases at the nasal root, 80% at the middle nasal dorsum, and 98.3% at the nasal tip.^6^

The impact of varying pressures on vascular imaging during real‐time ultrasound and differences among operators can significantly affect the quality and accuracy of the imaging results.

Different pressures applied during ultrasound scanning can influence the visualization of vascular structures. Excessive pressure may compress vessels, leading to distortion or even collapse, affecting the clarity and completeness of the images obtained. On the other hand, insufficient pressure may result in inadequate contact between the transducer and the skin, leading to suboptimal imaging quality and difficulty in identifying vascular structures.

Moreover, differences among operators, including their level of experience, technique, and proficiency in ultrasound imaging, can also impact the quality of vascular imaging. Experienced operators may have better spatial orientation and understanding of vascular anatomy, enabling them to obtain clearer and more accurate images.

The primary limitation of our study is its sample size, along with other challenges such as the impact of varying pressures on vascular imaging during real‐time ultrasound and discrepancies among different operators. Further validation of our findings could be achieved through additional multicenter studies involving diverse populations. The outcomes of this case series demonstrate that aesthetic enhancements were successfully achieved through an outpatient nasal filling procedure using a biodegradable product, guided by ultrasound imaging and adhering to anatomical and clinical patient standards.

## 5 CONCLUSION

Nasal filling, when executed with the appropriate technique following meticulous anatomical and cephalometric assessment, presents substantial advantages in various clinical scenarios. Formal education and practical experience are crucial for guaranteeing efficient and safe treatment, while the utilization of high frequency ultrasound can act as a pivotal tool in augmenting procedure safety. Real‐time ultrasound‐guided filler techniques have been developed to reduce the risk of vascular compromise confirming the distribution pattern of blood vessels. It's also crucial to visualize the cannula at the same moment as the vessels, even if the previous vascular mapping was performed.

## AUTHOR CONTRIBUTIONS

All authors conceived the present idea, designed the research study, analyzed the data, discussed the results, contributed essential reagents or tools and wrote the paper.

## FUNDING INFORMATION

The authors declare having received financial support by Rennova for the payment of an open access article. They also declare that there was no payment to the authors, physicians and patients involved in this study and that there was no other type of financial support.

## CONFLICT OF INTEREST STATEMENT

Bruna S. F. Bravo is a medical consultant of Merz, Rennova, Allergan, and Lóreal; the other authors declare no conflict of interest.

## ETHICS STATEMENT

The authors confirm that the ethical policies of the journal, as noted on the journal's author guidelines page, have been adhered to. All participants have signed the informed consent form and they participated in our study voluntarily. No ethical approval was required, reinforcing that it followed all the ethical principles of studies. The authors ensure that participants' rights are protected.

## INFORMED CONSENT

All study patients provided written informed consent before treatment.

## Supporting information


Figure S1.

Figure S2.

Figure S3.

Video S1.

## Data Availability

The data support the findings of this study are available from the corresponding author upon reasonable request.
